# Inefficient Processes and Associated Factors in Primary Care Nursing: System Configuration Analysis

**DOI:** 10.2196/49691

**Published:** 2024-09-30

**Authors:** Willi L Tarver, April Savoy, Himalaya Patel, Michael Weiner, Richard J Holden

**Affiliations:** 1 Division of Cancer Prevention and Control Department of Internal Medicine The Ohio State University Columbus, OH United States; 2 Health Systems Research Center for Health Information and Communication (13-416) Richard L Roudebush Veterans Affairs Medical Center United States Department of Veterans Affairs Indianapolis, IN United States; 3 School of Industrial Engineering Purdue University Indianapolis, IN United States; 4 Regenstrief Institute, Inc Indianapolis, IN United States; 5 School of Medicine Indiana University Indianapolis, IN United States; 6 School of Public Health Indiana University Bloomington, IN United States

**Keywords:** health information technology, mobile devices, nursing and nursing systems, outpatient care, SEIPS 2.0, work-system analysis

## Abstract

**Background:**

Industrywide, primary care nurses’ work is increasing in complexity and team orientation. Mobile health information technologies (HITs) designed to aid nurses with indirect care tasks, including charting, have had mixed success. Failed introductions of HIT may be explained by insufficient integration into nurses’ work processes, owing to an incomplete or incorrect understanding of the underlying work systems. Despite this need for context, published evidence has focused more on inpatient settings than on primary care.

**Objective:**

This study aims to characterize nurses’ and health technicians’ perceptions of process inefficiencies in the primary care setting and identify related work system factors.

**Methods:**

Guided by the Systems Engineering Initiative for Patient Safety (SEIPS) 2.0 model, we conducted an exploratory work system analysis with a convenience sample of primary care nurses and health technicians. Semistructured contextual interviews were conducted in 2 sets of primary care clinics in the Midwestern United States, one in an urban tertiary care center and the other in a rural community-based outpatient facility. Using directed qualitative content analysis of transcripts, we identified tasks participants perceived as frequent, redundant, or difficult, related processes, and recommendations for improvement. In addition, we conducted configuration analyses to identify associations between process inefficiencies and work system factors.

**Results:**

We interviewed a convenience sample of 20 primary care nurses and 2 health technicians, averaging approximately 12 years of experience in their current role. Across sites, participants perceived 2 processes, managing patient calls and clinic walk-in visits, as inefficient. Among work system factors, participants described organizational and technological factors associated with inefficiencies. For example, new organization policies to decrease patient waiting invoked frequent, repetitive, and difficult tasks, including chart review and check-in using tablet computers. Participants reported that issues with policy implementation and technology usability contributed to process inefficiencies. Organizational and technological factors were also perceived among participants as the most adaptable. Suggested technology changes included new tools for walk-in triage and patient self-reporting of symptoms.

**Conclusions:**

In response to changes to organizational policy and technology, without compensative changes elsewhere in their primary care work system, participants reported process adaptations. These adaptations indicate inefficient work processes. Understanding how the implementation of organizational policies affects other factors in the primary care work system may improve the quality of such implementations and, in turn, increase the effectiveness and efficiency of primary care nurse processes. Furthermore, the design and implementation of HIT interventions should consider influential work system factors and their effects on work processes.

## Introduction

To meet the rising demand for primary care services [1], the role of primary care nurses is becoming more complex and team-based [2]. Additional industry-wide changes that further complicate primary care nurses’ roles include greater autonomy in care management [3] and growing telehealth duties (eg, managing patients using videoconferencing and remote patient home monitoring) [4], in addition to in-person care. Mobile health information technologies (HITs) such as laptops, tablets, and smartphones, particularly among nurses, have been used to facilitate flexibility in documentation, communication, and other tasks that would typically take nurses’ attention away from their patients [5-7]. However, inefficient HITs may increase nurses’ work burden and lead to unexpected changes in their roles and the dynamics of their care teams [8].

A better understanding of the work system for nurses and their needs can inform the design, development, and successful implementation of these technologies [9]. The few identified systems-level studies involving primary care nurses have demonstrated the usefulness of such a perspective [10,11]; however, to our knowledge, research specifically on primary care nurses’ work systems is sparse. To better understand nursing tasks and processes and the work system specific to the primary care context, accounting for the various factors affecting nurses’ work is needed [12]. Incorporating work system factors, including people, technology, tasks, organizations, and environments [13], may lead to improvements in the design and implementation of HITs. Furthermore, while each of these constructs is uniquely important, assessing them individually fails to capture how these constructs interact with each other. Accurately eliciting and identifying the needs of this population and understanding the work system specific to the primary care context requires accounting for the various factors affecting nurses’ work [12], accounting that may be served well by a systems-level human factors perspective.

The objective of this study is to (1) characterize nurses’ perceptions of process inefficiencies in the primary care setting and (2) describe related work-system factors. The Systems Engineering Initiative for Patient Safety (SEIPS) 2.0 model provides a user-centered, systems-level view of work system structure, processes, and outcomes in health care and their relationships [13]. The SEIPS 2.0 model posits that the sociotechnical work system produces work processes which shape outcomes [13]. Understanding how these factors interact has important implications for the nurses’ workflow, and the implementation of interventions designed to aid them in completing tasks may influence their beneficial or adverse effects on clinical care. In addition, as more HITs are being introduced into the primary care setting [14,15], our findings will serve as an important step in understanding how to best design and implement these technologies to support primary care nurses.

## Methods

### Study Design

This was an exploratory study of the work system of primary care nurses and health technicians. Guided by SEIPS 2.0 [13], we conducted contextual interviews, observed work activities, used directed content analysis methods to identify findings, and used configural diagramming to organize and report the findings.

### Setting

Our study focused on ambulatory care settings. Recruitment and data collection occurred at 2 sets of primary care clinics in the Midwestern United States. Site 1 was an urban, tertiary care medical center in a large city; site 2 was a rural, community-based outpatient facility in a small town. At both sites, nurses and health technicians regularly interact with an electronic health record (EHR) system to complete nursing processes. At site 1, some clinics distributed laptop computers to nurses, while all other staff used desktop computers in staff workrooms and examination rooms. Site 1 was a participant in the US Department of Veterans Affairs (VA) Mobile Health Provider Program launched in 2014; through this program, over 12,000 Apple iPads have been distributed at more than 60 VA sites, though device usage has been reportedly less than expected [16]. At site 2, nurses and health technicians had open workrooms and used ruggedized portable computers (Panasonic Toughbook CF-H2). Docks for mobile devices were installed in workrooms and examination rooms.

### Recruitment

Convenience sampling was used to identify nurses and health technicians at the primary care clinics. In the clinics of this health care system, health technicians work under the supervision of registered nurses to maintain the documents and records used in primary care nursing processes. A list of eligible primary care staff providing care in the clinic was obtained. Staff members were contacted by email to solicit participation. Primary care nurses and health technicians were subsequently contacted in person to gain their consent to engage in the interview process.

### Conceptual Model

SEIPS states that a person (eg, health care professional) performs tasks in the clinical care setting that require various tools and technologies (eg, HITs). The use of these tools and technologies to perform these clinical tasks occurs within a physical internal environment governed by organizational conditions as well as a broader external environment [13]. These components make up the work system, interact with each other, and influence each other. Variations in how these components interact can be associated with workflow and health outcomes. Furthermore, SEIPS 2.0 introduces concepts of configurations and adaptations [13]. For example, with each of the work system factors that can interact with one another, the concept of configuration acknowledges that not all of these components are relevant to each process or situation. More specifically, configuration pertains to the subset of components and their interactions that are actually relevant to a particular process or situation [13]. According to SEIPS 2.0, adaptations refer to the changes that have been attempted to decrease the gap between actual and ideal performance [17].

### Contextual Interviews

We conducted semistructured contextual interviews among primary care nurses and health technicians. This method of interviewing allows researchers to observe and ask clarifying questions to participants while they are working [18]. Participants assume the role of the expert and are able to demonstrate tasks while working, which may also prompt the discussion of tasks that they may not consider important to the topic during a traditional interview. Researchers, on the other hand, assume the role of a student or apprentice, trying to understand the work process to identify ways of improving it or implementing interventions to address any underlying problems or challenges [19].

A semistructured interview guide (Multimedia Appendix 1) was created by the research team based on SEIPS 2.0 [13]. Interview topics included (1) nurses’ perspectives on process inefficiencies in primary care; (2) tasks that were considered frequent, repetitive, difficult, and related to inefficient processes; (3) the types of information needed to complete tasks; (4) the tools and technology needed to complete tasks; (5) organizational factors or policies that affect primary care nurses’ abilities to complete tasks; and (6) the use of mobile applications. Terms and their associated definitions were provided to participants before the interview to establish a shared understanding (more details in Table 1). To match the study’s focus on HIT, the task scope was limited to clinical and administrative information.

**Table 1:** Terms and definitions used during interviews with primary nurses and health technicians on process inefficiencies.

Term	Definition
Information-intensive tasks	Require reading, writing, or sharing information (eg, chart review).
Frequent tasks	Performed often or for each patient (eg, looking up patients’ contact information or reviewing discharge summaries).
Repetitive tasks	Tasks done repeatedly that should only be done once or not at all (eg, repetitious logins or clicks to access required information).
Difficult tasks	Tasks requiring large amounts of concentration to complete (eg, reviewing labs or determining trends in vitals).

A total of 6 nonclinical researchers (4 with previous interviewing experience, including coauthor HP and 2 volunteers) conducted interviews using a prepared interview guide (Multimedia Appendix 1). Early interviews were led by the 4 experienced staff members, with 1 or 2 other researchers serving as notetakers. These early interviews served as training for the volunteers. In later interviews, researcher roles were rotated to limit the influence of any single interviewer.

Each session was led by 1 interviewer and 1 note-taker. Interviews lasted approximately 45 minutes and were conducted either in the general practice setting of the participants or in a private room. Each interview was audio-recorded and transcribed. Transcripts were done through a contracted professional service. Staff research assistants corrected major transcription errors and removed personal identifiers.

### Analysis of Contextual Interviews

Initial qualitative analysis was done by 4 staff research assistants. All had previously served as interviewers. Each transcript was coded fully by 2 staff research assistants. Segments of data (eg, a phrase, sentence, or group of sentences) were coded iteratively. In the first iteration of coding, we identified work system components. We then identified processes performed by primary care nurses and whether they were perceived as frequent, repetitive, or difficult. Furthermore, 3 analysts (WT, AS, and HP) conducted a directed content analysis guided by the SEIPS 2.0 model to identify work system configurations related to frequent, repetitive, and difficult tasks for specific processes [20,21].

### Configuration Analysis

The interactions of the coded work system components and processes were used to identify work system configurations. Interactions are defined as segments of data assigned to 2 or more coded components. We reviewed key findings with emphasis on the participant quotes and descriptions of processes and tasks to identify the tasks identified as most influential for each process. Next, we independently defined work system configurations for each frequently reported process and met to discuss and resolve discrepancies. We created configural diagrams [13] of work system elements related strongly to the identified inefficient processes. We then identified misalignments among work system factors. We define misalignment as a mismatch between human and nonhuman (eg, environment, policies, etc) factors that may lead to a breakdown of processes. Based on findings from the configurational analysis, we characterized adaptations as workarounds (ie, current adaptations) or recommendations (ie, future adaptations).

### Ethical Considerations

This study complied with the American Psychological Association Code of Ethics and was approved by the Research and Development Committee at the Richard L. Roudebush Veterans Affairs Medical Center and the Indiana University institutional review board (protocol #1611241830).

## Results

Among the 51 eligible staff members who were contacted, 20 nurses and 2 health technicians participated in this study and completed interviews (Table 2). Most participants were female (19/22, 86%); most participants were White (17/22, 77%). Participants had a mean of 8.3 (SD 8.7, range 1-36) years of experience with their current health care employer and 11.9 (SD 8.9, range 1-30) years in their present role.

**Figure 1:**
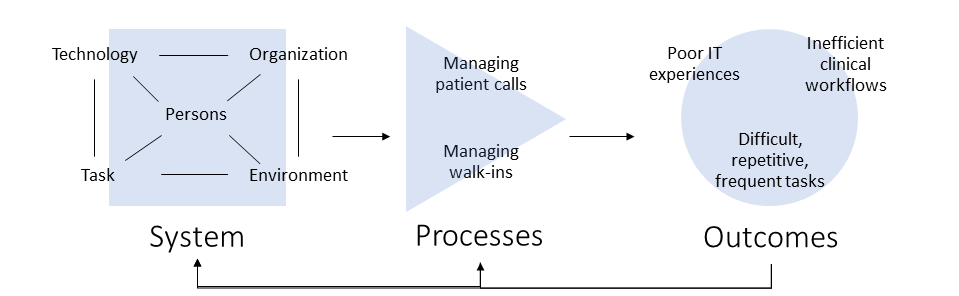
Application of SEIPS (Systems Engineering Initiative for Patient Safety) 2.0 to primary care nursing work systems, processes, and outcomes. Interviews with nurses and health technicians focused on inefficient processes and underlying work systems.

Based on our analysis, Figure 1 depicts the relationship among the nursing work system, processes, and perceived outcomes (Figure 1). Perceived inefficient workflows were associated with managing patient calls (ie, patient response calls) and walk-in patient processes. Related to these processes, participants reported managing notifications, documentation, and chart review as the most frequent, repetitive, and difficult tasks. These discussions highlighted both opportunities and potential barriers to the implementation of potential adaptations to HIT and policies for primary care nursing.

In the following sections, we report on the processes, their influential tasks, and the work system configurations, showing the most relevant components in each process.

**Table 2:** Demographics of participants at 2 primary care sites, one in an urban medical center (Site 1) and the other in a small community-based outpatient clinic (Site 2).

Characteristic		Both sites (N=22)	Site 1 (n=16)	Site 2 (n=6)
**Role, n (%)**				
	Registered nurse	14 (64)	10 (63)	4 (67)
	Licensed practical nurse	6 (27)	4 (25)	2 (33)
	Health technician	2 (9)	2 (12)	0 (0)
**Race, n (%)**				
	White	17 (77)	11 (69)	6 (100)
	Black	2 (9)	2 (19)	0 (0)
	Asian or Pacific Islander	3 (14)	3 (12)	0 (0)
**Gender, n (%)**				
	Female	19 (86)	14 (88)	5 (83)
	Male	3 (14)	2 (12)	1 (17)
Years in the role, mean (SD)		11.9 (8.9)	10.6 (8.6)	15.3 (8.8)
Years with current health care employer, mean (SD)		8.3 (8.7)	9.1 (9.8)	6.2 (3.3)

### Inefficient Nursing Processes

Managing patient calls and walk-in visits was perceived by participants as inefficient. For patient calls, contributors to inefficiency included repeated attempts to return patient calls and the EHR’s inability to track follow-ups. For walk-in processes, contributors to inefficiency included paper-based check-in and incomplete, self-reported patient information. We describe these in more detail below.

#### Managing Patient Calls

Some participants reported that they received 20 to 50 telephone calls daily from their site’s patient call center, which assists patients with various issues, such as scheduling multiple appointments and answering questions related to their care, medication, or paperwork. Patients also contact the call center to return missed calls. Other inbound calls came from patients sharing their frustrations, which participants reported as potentially time-consuming. Participants also received alerts from their EHR system that patients had called the call center. Along with using the EHR system to process alerts, participants also referred to the EHR when calling patients.

#### Managing Walk-Ins

Walk-in patients are patients who arrive at the clinic without an appointment. Participants noted that on a typical day, many of their patients were walk-ins. Nurses are required to see these patients regardless of how many patients they have already scheduled, and walk-in visits can often be time-consuming depending on the patient’s needs. Managing walk-ins involved triaging and scheduling patients. Triaging patients includes the gathering of important clinical data (eg, vital signs) and preparing the patient to see their primary care provider. Triaging time can vary, depending on the patient and the number of clinical reminders that need to be completed. Walk-in patients often enter the clinic for reasons that do not require an on-site physician evaluation, such as needing a medication refill or having minor aches and pains.

### Configuration: Misaligned Work System Factors

**Figure 2:**
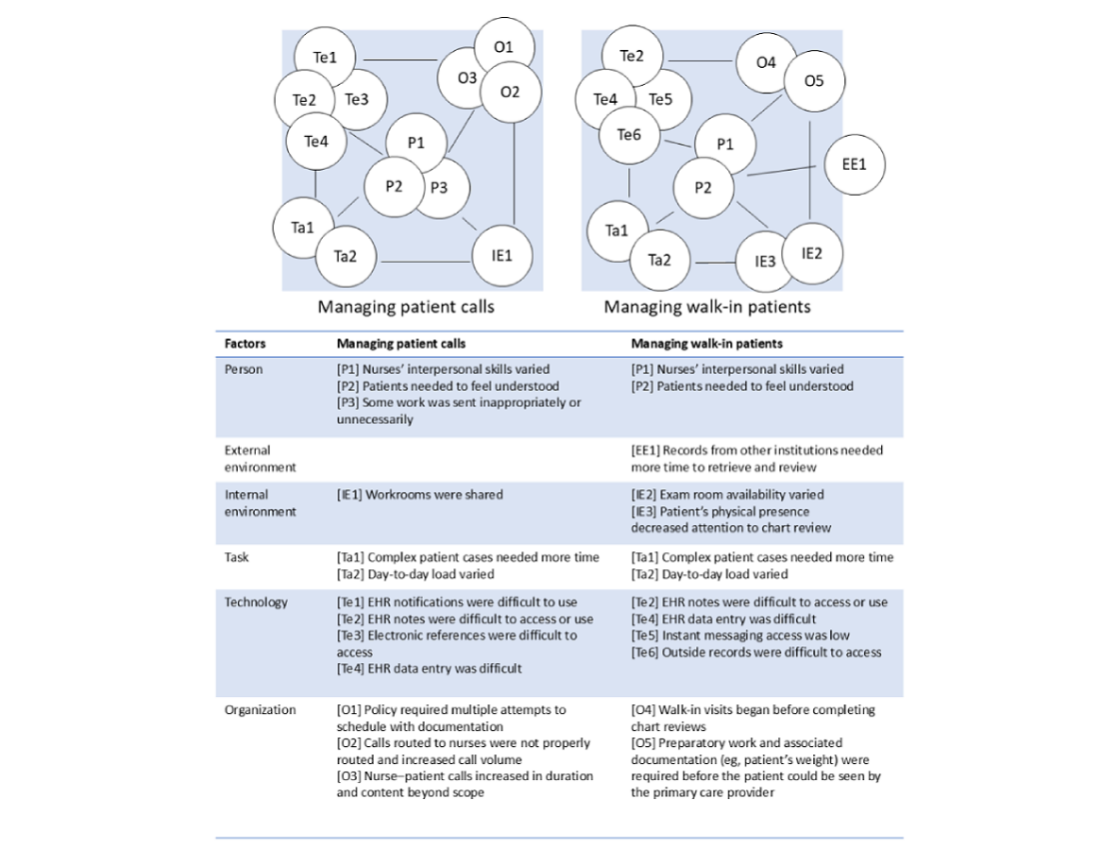
Application of SEIPS (Systems Engineering Initiative for Patient Safety) 2.0 to 2 primary care nursing work processes: managing patient calls and managing walk-in patients. Factor numbers are for identification and do not imply any order. Ta: Task; Te: Tools and technology; P: People; O: Organization; IE: Internal environment; EE: External environment.

Using SEIPS 2.0, we explored the work system configuration of the 2 aforementioned nurse processes, managing patient-response calls and walk-in patients. Participants reported these processes comprised the most frequent, repetitive, and difficult tasks. For each process, we explored the corresponding work system configurations and relevant factors. In Figure 2, we depict the configuration diagrams for both processes.

The following subsections further elaborate on each of the work system factors.

#### Person

Attitudes toward mobile HIT were shaped by experience with other technologies, including computers on wheels. Some participants viewed the paper as a facilitator of their work-related tasks and favored paper use over mobile technology or applications. Issues such as lack of time, susceptibility to interruption, and inability to take data to patients in nonexamination room spaces encouraged the use of the person’s memory skills or notes written on a piece of paper to transfer necessary patient information between various clinical spaces. Other issues related to other staff members, for example, a recurring issue from participants was that the call center does not properly triage patient calls made to the call center.

#### Environment: Internal and External

Notable environmental differences occurred across the 2 sites, although only a few tasks were affected by these differences. For example, triaging walk-ins varied among clinics. When nurses and health technicians had their own rooms, patients were triaged and seen by the physicians in that room. However, for nurses on 1 team, triaging was completed in 1 room, and then patients could be transferred into the physician’s examination room. Thus, for nurses on this team, triaging required finding rooms that were open and contained the right patient education pamphlets.

Participants described the “walk-in pickup,” which involves meeting walk-in patients in waiting areas to assess the presentation of symptoms in order to triage the patients quickly and effectively. This method was complicated by initial conversations occurring in nonprivate locations, which limited the level of detail that could be discussed, and by the inability to access medical records electronically during these conversations.

#### Tasks

Across the 2 processes, participants denoted how complex patient cases needed more time and how the day-to-day load varied. Regarding managing patient calls, participants described frequent tasks, as the call center directs all patient calls to primary care nurses regardless of individual patient needs. Furthermore, participants considered certain tasks that they completed on these calls to be repetitive, including patient education. Some participants indicated that they were largely repeating the information they had provided to patients during previous appointments, which patients had forgotten or were struggling to explain to family members. In addition, many calls come from patients trying to return nurses’ missed calls; the call center team is unable to relay any information or schedule appointments for these patients. These instances often resulted in multiple unnecessary back-and-forth phone call exchanges. Participants defined this task as difficult due to the extensive amount of time it took to prioritize the list of calls and related alerts.

Participants also perceived chart review for these patients to be particularly challenging, typically consisting of a review of recent notes, laboratory results, orders, imaging reports, medication lists, or other information to familiarize staff members with a patient’s background and recent medical history. Reviews were considered at the following 2 levels. (1) Flash review, which refers to the quick review of medical records in response to the initial question, “I wonder why they’re coming?” (2) In-depth review, which refers to the more detailed, investigative review of medical records, typically required for care management. The in-depth review may involve “piecing together” information from a chronological series of notes to discover the narrative patient history and the synthesis of information gathered from different types of notes, tabs, or displays within the EHR. Both types of reviews are complicated when staff members are not familiar with patients (eg, new patients and those from other staff members’ panels) and when interruptions occur frequently.

#### Tools and Technology

Across sites, nurses described problematic aspects of the existing mobile technology. Some participants at site 2 characterized the ruggedized portable PCs as being bulky and useless. Although portable PCs eliminated the need to log in to multiple desktop PCs, mobile devices lost access to the EHR when connecting to or disconnecting from docking stations. Each transition caused the PC to swap between wired and wireless network connections. The connection swap, in turn, disconnected the user from the EHR, resulting in the loss of any unsaved data entered by the user. Instead of providing continuous information interaction and access to the EHR as expected, the portable PCs were usable mainly when docked. This limitation disappointed nurses; mobile devices were used more as luggable desktop computers than for facilitating efficient data collection and communication as expected. Furthermore, at site 1, where mobile technology was distributed and could be used voluntarily, no participants reported it useful for clinical tasks.

#### Organization

Furthermore, 2 organizational policies influenced the use and usefulness of mobile HIT across the sites. The first was the patient flow policy to limit unnecessary room changes during a health care visit. At site 1, patients move between stations when nurses need to measure vital signs and collect specimens. These stations were often near the nurses’ desks, which decreased the need for mobile HIT. At site 2, a policy aimed at reducing patient flow was implemented that required various health care providers, including nurses, to meet the patient in the examination room. This increased the need for nurses to review and document information (eg, vital signs) in examination rooms and hallways away from their desks. In addition, an organizational policy was in place at site 1 that made the use of mobile HIT voluntary. At site 2, the use of mobile HIT was mandated; it was this mandate that accounted for the sustained use of mobile HIT.

We identified work system configurations that contributed to participants’ perceptions of inefficient processes. Table 3 displays recurring topics and sample excerpts of configuration details anchored by processes’ frequent, repetitive, and difficult tasks. The process of managing patient calls was associated with several tasks that were identified as frequent, repetitive, and difficult. For example, handling patient calls that were inappropriately assigned was identified as both frequent and repetitive. The relevant factors for this process included person factors (ie, patients’ need to feel understood, which may be difficult when the patient is assigned to the wrong person), organization factors (ie, this may result in calls increasing in duration and content beyond the scope of the recipient’s work), and task factors (ie, the day-to-day load for handling inappropriately assigned calls can vary).

**Table 3:** Primary care nurses’ and health technicians’ work system configuration details anchored by processes’ frequent, repetitive, and difficult tasks.

Process and task			Representative quote^a^		SEIPS^b^ 2.0 work system configuration
**Managing patient calls**					
	Handling patient calls that were inappropriately assigned (Frequent)	“They’re calling about an issue that is out of my control, they’re very upset, they didn’t want their methadone to go away, their methadone is gone, and they want to talk about 30 minutes. That’s a real time-waster for me because, you know, that’s, I realize they want an outlet, and they’re frustrated, but for me, I can’t help them, and they’re just getting more worked up. Things like, a lot of calls like that where there’s really just nothing to do and all they really want to do is talk to someone, but I’m not getting anything done.”		[P^c^2] Patients needed to feel understood [O^d^3] Nurse–patient calls increased in duration and content beyond scope [Ta^e^2] Day-to-day load varied	
	Handling patient calls that were inappropriately assigned (Repetitive)	“The patient calls and the call center relays the message to me if it needs to come to Primary Care. Sometimes it doesn’t need to come to Primary Care. Sometimes what usually happens though is I will call the patient back to speak with the patient. I can’t get a hold of the patient so then they’re calling the call center again. Well, at that point I have a patient in my office. I can’t take the phone call. We’re doing that all day long. We’re playing phone tag constantly. Just very redundant.”		[P1] Nurses’ interpersonal skills varied [O3] Nurse–patient calls increased in duration and content beyond scope [Ta2] Day-to-day load varied	
	Managing notifications (Repetitive)	“Oh, there are a lot of view alerts where they send them to multiple people, and a lot of times, they could just come to me. As soon as I see it, I’ll do it. But there again, now 2 or 3 other people have to look at it and see if it’s been done. That’s kind of redundant.”		[P3] Some work was sent inappropriately or unnecessarily [Te^f^1] EHR^g^ notifications were difficult to use [Ta2] Day-to-day load varied	
	Scheduling appointments with patients when they are not in the office (Repetitive)	“They want us to continue trying to contact these patients and to me, it just seems redundant. If the patient, wanted an appointment, they would’ve called and scheduled you know, so it seems like a waste of time when I’ve got 40 people that I’m trying to call and I have to put a note here, I have to chart it over here, I have to delete the recall.”		[O1] Policy required multiple attempts to schedule with documentation [Te4] EHR data entry was difficult [Ta2] Day-to-day load varied	
**Managing walk-ins**					
	Checking in walk-ins (Frequent)	“There’s somewhere anywhere from 8 to 12 patients scheduled a day and we’ve got to get them ready to see the provider which can take some time. It can take anywhere from 10 minutes, or it might take 30 minutes to check in some of these patients so that takes up the majority of the time.”		[Te4] EHR data entry was difficult [Ta1] Complex patient cases needed more time [Ta2] Day-to-day load varied	
	Quick patient assessment and triage (Frequent)	“Because I may only get 2 or I may get 7 but they take up a great deal of time and they walk in and you need to stop whatever you’re doing and go out to them right then.”		[O4] Walk-in visits began before completing chart reviews [Ta2] Day-to-day load varied	
	Chart review of external medical records (Repetitive)	“It’s just time consuming to kind of sit and look at all that, especially if you have a patient that maybe came from the outside VA...they might say you know “well I had all this done at the other VA and so kind of trying to pull all the VistA Web [health information exchange service] stuff and look at that is kind of, definitely that’s time consuming.”		[O5] Preparatory work and associated documentation (eg, patient’s weight) were required before the patient could be seen by the primary care provider [Te6] Outside records were difficult to access [EE^h^1] Records from other institutions needed more time to retrieve and review [Ta2] Day-to-day load varied	
	Chart review for new patients with little time before patient visit (Difficult)	“Sometimes the walk-ins can be difficult because it might not be your patient if you’re covering for someone. You don’t know the patient at all and you’re trying to piece it all together, because you can’t just go to the walk-in doctor and say they’re here, they say their head hurts. That’s not going to fly, you know, so it’s kind of a lot of maybe 15-20 minutes in their chart, and if the patient is in there while you’re doing it, that’s kind of distracting, because they don’t understand that you don’t know them and you need to review their chart, so they just take off with their current situation assuming you know their background often, so yeah....”		[P2] Patients needed to feel understood [O4] Walk-in visits began before completing chart reviews [Te2] EHR notes were difficult to access or use [IE^i^3] Patient’s physical presence decreased attention to chart review [Ta1] Complex patient cases needed more time	
**Managing patient calls; managing walk-ins**					
	Chart review before the patient visit (Difficult)	“At least if it’s a phone call, I can see the message and why they’re calling. I can review the chart. If I need help before calling them back, I can go ahead and get that. When you’re sitting in front of somebody and they say well, I’m coughing and I have a headache. I’m having to do all of this in real time.”		[P1] Nurses’ interpersonal skills varied [O4] Walk-in visits began before completing chart reviews [IE3] Patient’s physical presence decreased attention to chart review [Te2] EHR notes were difficult to access or use [Ta1] Complex patient cases needed more time	
	Patient education and chart review (Difficult)	“It takes a lot of time because it’s a lot of educating the patients and you know looking back, what was done before, going through labs, meds. You’ve got to go through side effects of meds and assess everything completely. So, those can be a little bit time consuming… ”		[P1] Nurses’ interpersonal skills varied [Ta1] Complex patient cases needed more time [Te2] EHR notes were difficult to access or use	

^a^Relevant work system elements are listed for the representative quotation. The corresponding task may include factors not listed here.

^b^SEIPS: Systems Engineering Initiative for Patient Safety.

^c^P: People.

^d^O: Organization.

^e^Ta: Task.

^f^Te: Tools and technology.

^g^EHR: electronic health record.

^h^EE: External environment.

^i^IE: Internal environment.

### Adaptations: Perceived Adaptability and Recommendations

Work system components “Tools and technology” and “Organization” were associated with the most misaligned factor configurations (Table 3). In addition, these factors were perceived to be the most adaptable among participants. Some reported adaptations were workarounds, while other adaptations were recommendations for unmet needs. Participants did not describe or discuss recommendations related to the remaining work-system components (People, Environments, and Tasks).

### Workarounds

Primary systemwide adaptations created process inefficiencies, leading to secondary localized adaptations in the form of user workarounds. At site 2, participants reported that the policies for patient-centered flow and dockable PCs, taken together, limited their EHR review and charting to the times that their PCs were docked. Their interim storage needs were met using paper notes, which also addressed the risk of data loss from EHR disconnections. Paper notes were shared with physicians, who could then review and add to the notes before the notes were entered into the EHR after the visit.

Site 1’s walk-in policy, combined with the physical layout of the clinic, was also linked to the use of paper notes. One site 1 clinic created a paper intake form for patients to self-report the reason for their visit and the reason for walking in instead of alternatives (eg, making an appointment or refilling medications through the patient portal). For health technicians, paper notes indicated double documentation: for example, the patient’s weight would be written while in the corridor by the weight scale, then reentered into the EHR afterward. Using a different workaround, the “walk-in pickup” described in the previous section, participants balanced their need for intake information with their need to manage each patient’s expectations about when they would be seen. This approach necessitated a chart review in which staff looked for information to aid triaging, including the time since the patient’s last visit and the frequency and nature of the patient’s previous walk-in visits.

At site 1, to work around inefficiencies with patient calls, 1 participant reported using their appointment scheduler software to track outbound follow-up calls about lab results. However, these appointments appeared in the patient-facing portal, which sometimes confused patients who were not expecting such calls.

### Recommendations

Recommendations included changes to “tools and technology.” Among the participants’ suggestions to improve walk-in management were new tools for symptom self-reporting and triage. To improve the check-in process for walk-in patients, 1 recommendation from a participant was a patient-facing technology for collecting patients’ descriptions of their health issues (eg, symptoms of congestive heart failure). Another participant’s suggestion was a personalized display of the expected waiting time, encouraging patients with less serious ailments to consider scheduling an appointment or requesting information about alternative ways to address their medical concerns. For themselves, participants sought a method to view relevant patient trends, minimizing the need for rushed chart review. Many participants wanted call center staff to offer greater mediation between themselves and patients. Currently, back-and-forth communication is needed to understand a patient’s concerns or issues. More and better training resources could be made available.

## Discussion

### Principal Findings

In this study, we described primary care nurses’ work system configurations associated with inefficient processes, misaligned work system factors, and adaptations to guide future interventions. Managing patient calls and managing walk-in patients were inefficient processes. The results from our work system analysis defined nursing tasks associated with each process that was described as frequent, repetitive, or difficult among primary care nurses and health technicians. In addition, we applied SEIPS 2.0’s configuration concept [13] to illustrate subsets of work system factors that were associated with workflow inefficiencies. With the model’s adaptation concept, we characterized the propagating, negative impacts of changes to a work system component. Some adaptations were made by health care workers in the form of workarounds, with varying success, while other adaptations were recommended.

### SEIPS 2.0 Application to Primary Care Nursing Processes

Although SEIPS 2.0 has been used to identify tasks associated with decreased work ability among inpatient nurses [22,23], to our knowledge, this is among the first applications of SEIPS 2.0 specifically to primary care nursing processes. The original SEIPS framework [24] has been identified as a means of describing and evaluating processes in primary care by taking into account the complex, interconnected socio-technical aspects found in the health care system [25]. Lagisetty et al [26] organized their systematic review of primary care opioid use disorder interventions using SEIPS 2.0’s concepts of work system factors, processes, and outcomes [13]. Robertson et al [11] used SEIPS 2.0 [13] to identify barriers and facilitators to integrating practice guidelines to reduce under-5 mortality in a primary care clinic in Malawi; their recommendations addressed mostly organizational factors. More recently, Werner et al [20] used SEIPS 2.0 configuration diagrams [13] to illustrate work barriers and facilitators within work system configurations for older adults’ transitions between emergency department to home. McCormack et al [27] used SEIPS 2.0 to identify facilitators and barriers to referrals between primary and specialty care services.

### Misaligned Work System Factors

The misalignment of tasks, organization, and technology factors was described among multiple configured sets of work system factors. Patient flow policies introduced more nomadic or mobile aspects and interruptions to primary care nursing processes. Other studies have defined telephone calls and conversations as the most common sources of interruptions for nurses and health technicians [28]. In this study, we refer to these as unscheduled tasks. In workflows, increased movement of primary care nurses increased the amount of missed patient phone calls, alerts about missed patient phone calls, and associated patient voicemails. Furthermore, increased presence in hallways and rooms appeared to yield more impromptu conversations. The implemented mobile HIT to support this patient flow was limited by poor connectivity to the network making data transfer difficult, and possible data loss when undocking mobile devices. Experiencing high levels of process discontinuity predisposes health care staff to make errors [29,30]. Processes with unscheduled tasks introduced work fragmentation or a break in continuous work activity. Despite demonstrated resilience against interruptions [31], unscheduled tasks increased nurses’ and health technicians’ cognitive load and decreased their ability to recall information needed to complete task switching effectively [32]. Minimizing unnecessary interruptions is particularly important in the health care context, where failing to complete tasks can have adverse effects on health outcomes and patient safety.

### Adaptations: Feedback and Indicators of Gaps

Based on SEIPS 2.0, the inefficiencies described in our study can be indicators of gaps in performance, quality, or patient safety [13]. Whether initiated or recommended by nurses, the adaptations identified in this study were mostly reactive to new policies recently implemented. These adaptations indicated gaps in HIT performance and quality of nursing processes, which may be linked to patient, nurse, or organizational outcomes [33]. Previous studies have denoted that traditional or routine clinical quality indicators do not always include measures for HIT-related aspects of workflow, such as usability [27,34]. Poor usability of HIT is associated with various types of adaptations, often referred to as workarounds [35,36]. For example, the new policies and HIT (ie, planned adaptations) were implemented to improve patient flow and access to primary care services. Yet, nurses described the generation of new gaps or inefficiencies that propagated throughout the work system, which we characterized as misalignments. Based on their experiences, nurses were able to identify inefficiencies, implement workarounds (adaptations to the patient flow adaptation), and suggest recommendations for future adaptations. While other interpretations of workarounds vary [33,37], SEIPS 2.0 represents these types of adaptations as feedback. Without this type of feedback, monitoring such dynamic complex work systems, important indicators and gaps would not be recognized, and efforts for continuous improvement would be hindered.

### Future Design and Implementation Strategies

Studies like this are foundational to future interventional research. Future design and implementation of interventions for these and similar nursing processes are warranted as health care use increases and diversifies with same-day and virtual visits, which have similar aspects and common tasks with the inefficient processes identified in this study. Due to the interconnectivity of work systems, adaptations focused on a single component or subset of components will affect other components. Whether the adaptation is planned or unplanned, the lack of consideration for all the components in the work system increases the potential for negative, unintended consequences. Without work system analyses, the implementation of interventions can negatively affect clinicians, patients, and organizational outcomes. Similar problems in other clinician groups have been addressed by incorporating system-level design and implementation of HIT [38]. Yet, varying institutions have unique needs or processes that demand different solutions. For example, the distribution of mobile devices without apps that are tailored to our participants was insufficient to meet their needs. Therefore, understanding the personalized challenges of an institution warrants a systems-level analysis. Our findings provide a necessary first step in the development and integration of future health information technologies that improve the efficiency of health care delivery by supporting frequent, difficult, and repetitive tasks for nurses. In addition, this research shows that for interventions, including health information technology implementation, to be successful, implementers must also account for work system factors such as organizational policies. Based on our findings, the major opportunities for adaptations are related to workflow policies and supporting health information technologies for primary care nurses.

Since the completion of our study, the VA’s Office of Connected Care is supporting an increasing number of provider- and patient-facing mobile apps, including task-specific apps [39]. Providers currently have access to a variety of task-specific apps for mobile computing through the VA App Store [39].

### Strengths and Limitations

A strength of this study is that it uses a human factors approach to identify major contributors to inefficiencies at the systems level. This study also has several limitations. A limitation of this study is that the observations and interviews were performed in clinics belonging to 1 integrated health care institution. Convenience sampling may have introduced biases in participants’ reporting of work processes and barriers. Our findings may not entirely transfer to other health care systems and settings. Therefore, more attention should be given to aiding in the design and development of user-centered apps in different settings with different work system configurations. Similarly, while we identified notable differences between the 2 sites used for this study, we did not further assess how those differences contributed to our results. Finally, this study focused only on the potential of mobile HIT as a solution to process inefficiencies. As a result, this may have elicited confirmation bias.

### Conclusion

Nurses and health technicians perceived that the implementation of new policies and technologies contributed to inefficiencies in nursing workflows across ambulatory settings. A system analysis was an effective method for identifying configured subsets of work system factors associated with perceived gaps in nursing processes. Furthermore, the configuration and adaptation concepts in the SEIPS 2.0 framework aided in the characterization of adaptations to inform future research and interventions. To identify both potential consequences across work system components and nursing processes, system analyses are warranted for the design, implementation, and evaluation of organizational policies or HIT.

## Appendix

Multimedia Appendix 1 Interview guide.

## Abbreviations

EHR: electronic health record
HIT: health information technology
SEIPS: Systems Engineering Initiative for Patient Safety
VA: US Department of Veterans Affairs

## Data Availability

The data sets generated during and/or analyzed during this study are not publicly available due to the presence of personally identifiable information but are available from the corresponding author on reasonable request.
