# Supplementary material for: Inefficient Processes and Associated Factors in Primary Care Nursing: System Configuration Analysis
Source: JMIR Hum Factors. 2024 Sep 30;11:e49691. doi: 10.2196/49691 (PMC11474133; doi:10.2196/49691)
Supplement: Multimedia Appendix 1 [file humanfactors_v11i1e49691_app1.docx]

**Interview guide**

| **#** | **SEIPS Construct** | **Interview Question** |
| --- | --- | --- |
| 1 | Task | What would you say is/are your most FREQUENT task(s)? Please explain. |
| 2 | Task | What would you say is/are your most REPETITIVE task(s)? Please explain. |
| 3 | Task | What task(s) would you say is/are most DIFFICULT? Please explain.  [Then, for each task identified above, ask the following:] |
| 4 | Environment | Was this task mobile or performed away from your desk/office? If so, where was it performed? |
| 5 | Task | What types of information do you need to complete this task? |
| 6 | Tools and technology | What tools and technology do you use to complete this task? |
| 7 | Person | With whom do you communicate with to complete this task? |
| 8 | Organization | In what ways, if any, do organizational factors (for example: work schedules, work culture, management, and training) or policies affect your ability to complete this task? Using mobile apps? |
| 9 | Task and Tools and technology | How could mobile applications and technology help support this task? |
